# Stimulus rate–dependent selective prolongation of the I–III interpeak latency during intraoperative total intravenous anesthesia

**DOI:** 10.1016/j.cnp.2026.10.011

**Published:** 2026-11-04

**Authors:** Yoshinori Higuchi, Shigeki Nakano, Shinichi Origuchi, Satoru Kurihara, Tatsuma Matsuda, Kentaro Horiguchi

**Affiliations:** Department of Neurological Surgery, Chiba University Graduate School of Medicine, Japan

**Keywords:** Auditory brainstem response, Interpeak latency, Stimulus rate, Brainstem conduction, Linear mixed-effects model, Intraoperative monitoring

## Abstract

**Objective:**

Stimulus rate (SR) affects auditory brainstem response (ABR) latencies; however, its segment-specific effects under standardized intraoperative total intravenous anesthesia (TIVA) remain incompletely characterized. We investigated whether SR differentially affects early and late brainstem conduction segments.

**Methods:**

Intraoperative ABRs were recorded in 23 patients undergoing microvascular decompression under standardized propofol–remifentanil TIVA. SRs of 17, 29, 37, and 43 Hz were applied in a standardized sequence. Interpeak latencies (IPLs) for the I–III and III–V segments were analyzed using linear mixed-effects models to distinguish within-patient SR effects from between-patient variability. Fixed-effect slopes and 95% confidence intervals were estimated.

**Results:**

Although the absolute wave V latency increased with increasing SR, the III–V interpeak interval remained unchanged. The I–III IPL showed a significant positive association with SR when SR was modeled as a continuous variable (β = 0.00521 ms/Hz, 95% CI 0.00307–0.00735, *p* < 0.001), corresponding to an approximately 0.052-ms prolongation per 10-Hz increase. The III–V IPL was not significantly associated with SR (β = 0.00033 ms/Hz, 95% CI −0.00201 to 0.00267, *p* = 0.782). Individual trajectories showed a consistent effect in the early segment, with no systematic trend in the late segment.

**Conclusions:**

Under standardized TIVA conditions, SR selectively prolongs conduction time in the early brainstem segment (I–III), while showing a limited observable effect in the later segment (III–V). This rate-dependent pattern was consistent across patients over the tested SR range.

**Significance:**

Recognition of segment-specific SR-dependent modulation may improve the interpretation of intraoperative ABR monitoring and support the concept of a rate-sensitive processing stage in early auditory brainstem pathways.

## Highlights

• High rates selectively prolong early conduction during total intravenous anesthesia.
• Late conduction shows little rate dependence during total intravenous anesthesia.
• Recognizing early latency shifts may guide intraoperative auditory brainstem testing.

## 1 Introduction

Auditory brainstem responses (ABRs) provide a temporally precise measure of sequential neural activation along the auditory pathway and are widely used in both diagnostic neurophysiology and intraoperative monitoring. Stimulus rate (SR) is a key recording parameter that directly affects waveform morphology, latency measures, and signal-to-noise performance. Conventional clinical guidelines recommend relatively low stimulation rates (5–12 Hz) to minimize adaptation effects and preserve waveform stability (American Clinical Neurophysiology Society, 2006).

However, growing evidence suggests that SR-related changes in ABR components reflect neural adaptation and synaptic recovery kinetics rather than a uniform slowing of axonal conduction. Experimental studies have demonstrated that increasing the repetition rate systematically prolongs ABR peak latencies and may also alter amplitudes in a component-specific manner (Dzulkarnain et al., 2020; Holt and Ozdamar, 2016). Importantly, these rate effects are not evenly distributed across brainstem generators, supporting the concept of segment-specific vulnerability within early auditory relays (Holt and Ozdamar, 2016).

ABRs are known to be influenced by stimulation rate (SR), which affects both the latency and amplitude of evoked waveforms. Increasing SR typically results in prolonged wave latencies, particularly for later components such as wave V, accompanied by reduced amplitude. These rate-dependent changes have been consistently demonstrated in both classical and contemporary studies and are generally attributed to neural adaptation and limited recovery time within the auditory pathway (Starr and Achor, 1975; Lasky, 1997).

Importantly, the magnitude of SR-related latency shifts is not uniform across the auditory pathway. Earlier components, such as wave I, are relatively resistant to SR changes, whereas later components, including waves III and V, exhibit greater susceptibility, reflecting the increasing contribution of polysynaptic transmission and temporal integration at higher levels of the brainstem (Stockard et al., 1978; Don et al., 1994).

In the intraoperative setting, higher SR paradigms have been proposed to shorten acquisition times and improve monitoring responsiveness. Joo et al. reported that a stimulation rate of 43.9 Hz with 400 averaged trials enabled reliable ABR acquisition within approximately 9 s. Using a wave V amplitude loss of >50% as the warning criterion, they also found a significant reduction in postoperative hearing impairment (Joo et al., 2016). Notably, substantial prolongation of wave V latency was observed under high-SR conditions without permanent postoperative deficits, suggesting that latency-based warning thresholds may need to be reinterpreted when high-rate stimulation protocols are used.

Despite these advances, the magnitude and segmental distribution of SR-dependent latency shifts under standardized total intravenous anesthesia (TIVA) have not been fully characterized. Distinguishing cumulative synaptic delays within early brainstem relays from latency shifts affecting more rostral pathways is important for interpreting interpeak latency (IPL) measures during high-rate monitoring and for balancing rapid acquisition with physiological interpretability.

Therefore, we aimed to model SR-dependent modulation of absolute wave latencies and segmental IPLs using linear mixed-effects analysis under standardized TIVA conditions. We hypothesized that the early conduction segment (I–III IPL) would be more sensitive to SR than the later segment (III–V IPL), consistent with cumulative synaptic adaptation within proximal auditory brainstem relays rather than global slowing of conduction.

## 2 Methods

### 2.1 Study design and ethics

This retrospective observational study analyzed intraoperative ABR recordings routinely acquired during microvascular decompression (MVD) procedures. No additional ABR recordings or changes in intraoperative management were performed specifically for the present study. Preoperative hearing status was assessed using pure-tone audiometry. The study protocol was approved by the Ethics Committee of Chiba University Hospital (Approval No. HK202510–17). Written informed consent was obtained from all participants. This study was conducted in accordance with the principles of the Declaration of Helsinki.

### 2.2 Anesthesia protocol

All procedures were performed under a standardized TIVA protocol to minimize the confounding effects of volatile anesthetics on ABR waveforms. Anesthesia was induced and maintained using target-controlled infusion of propofol and remifentanil. The target effect-site concentration of propofol was maintained at 2.5 ± 0.4 μg/mL (mean ± Standard deviation [SD]), with a bispectral index (BIS) of 44.74 ± 7.62. During ABR recording, the depth of anesthesia was monitored using the BIS and maintained at 44.7 ± 7.6. Mean arterial pressure (65.0 ± 8.9 mmHg) and core body temperature (36.4 ± 0.4 °C) were maintained within stable physiological ranges throughout data acquisition. All ABR recordings were obtained immediately before surgical incision.

### 2.3 ABR acquisition and recording protocol

All ABR recordings analyzed in this study were obtained before skin incision. Intraoperative ABR monitoring was performed using the Neuromaster G1 system (MEE-2000; Nihon Kohden, Tokyo, Japan). Disposable electrodes were placed on the mastoid (reference), low forehead (ground), and vertex (Cz) (measurement channel). Stimuli consisted of 0.1-ms rarefaction clicks delivered monaurally to the operative ear via insert earphones. All ABR waveforms were recorded before the surgical procedure. The latencies of waves I, III, and V were determined by visually identifying their respective peaks. Amplitudes were defined as the instantaneous voltage at each peak latency relative to the 0.0 μV baseline. All measurements and peak identifications were performed by a trained intraoperative monitoring technician and a board-certified physician in intraoperative neuromonitoring certified by the Japanese Society of Clinical Neurophysiology.

As part of routine intraoperative ABR monitoring at our institution, recordings were obtained under eight predefined acquisition conditions before skin incision, comprising four SRs (17, 29, 37, and 43 Hz) and two averaging counts (200 and 500 sweeps). The SRs of 17, 29, 37, and 43 Hz were selected to span a clinically practical range for intraoperative ABR monitoring. Joo et al. previously demonstrated the feasibility of high-rate intraoperative ABR monitoring at 43.9 Hz; accordingly, 43 Hz was selected as the practical upper SR in the present protocol. Prime-number SRs were used in accordance with our routine intraoperative monitoring practice. These rates were therefore selected for their clinical applicability rather than as equally spaced experimental levels. These recordings were acquired for clinical monitoring purposes to assess waveform stability and acquisition efficiency and were not performed prospectively for the present research study. The current study retrospectively analyzed these routinely acquired recordings. SRs were tested in ascending order from low to high, with at least a 1-min interval between the end of one recording and the start of the next. Baseline ABR parameters were defined as those obtained under the lowest SR (17 Hz) and highest averaging count (500 sweeps) condition. The 17 Hz condition was designated as a baseline for descriptive comparison because lower SRs provide more stable waveforms with minimal adaptation.

The absolute latencies and peak-to-trough amplitudes of waves I, III, and V were measured. IPLs for segments I–III and III–V were calculated to evaluate conduction times through the early (distal auditory nerve to cochlear nucleus/superior olive) and later brainstem pathways, respectively.

### 2.4 Statistical analysis

For each patient and recording condition, the latencies of waves I, III, and V were measured directly from the recorded and averaged ABR waveforms, and the I–III and III–V IPLs were calculated from these measured values. These observed latency and IPL values constituted the dependent variables used in the statistical models; no model-generated or simulated ABR data were used.

Linear mixed-effects models with a random intercept for each patient were fitted to account for repeated measurements within patients. SR was modeled as a continuous variable or categorical factor, and averaging count was included as a covariate. The β coefficients reported from these models represent fixed-effect estimates of the association between SR and the observed ABR latency measures.

Statistical analyses were performed in Python (version 3.11; Python Software Foundation, Wilmington, Delaware, United States). Data handling and descriptive statistics were performed using pandas. Linear mixed-effects models with a random intercept for each patient were fitted using the statsmodels package to account for repeated measurements within patients. Model parameters were estimated by restricted maximum likelihood. SR was modeled both as a continuous variable (per 1-Hz and 10-Hz increases) and as a categorical factor. Averaging count was included as a covariate. Fixed-effect estimates and 95% confidence intervals (CIs) were obtained. Patient-specific slopes were derived by fitting a simple linear regression model for each patient, using SR as the independent variable and ABR latency or IPL as the dependent variable. The resulting slope represented the estimated latency change per 1-Hz increase in SR within each patient. Because only four stimulus-rate levels were tested, these slopes were interpreted as descriptive indices of individual rate sensitivity rather than primary inferential statistics. As a supplementary analysis, overall categorical effects of SR were evaluated using likelihood-ratio tests comparing nested mixed-effects models fitted by maximum likelihood.

Statistical significance was defined as *p* < 0.05. Continuous variables are reported as mean ± SD or 95% CIs, as appropriate. As a sensitivity analysis for potential nonlinearity, a quadratic SR term (SR^2^) was added to the mixed-effects model, and model fit was compared with that of the linear model using a likelihood-ratio test.

## 3 Results

The study included 23 patients with a mean age of 56.8 ± 13.9 years; 13 (56.5%) were men and 10 (43.5%) were women. Patient and intraoperative characteristics are summarized in Table 1. All recordings were obtained under standardized TIVA conditions, with physiological parameters maintained within stable intraoperative ranges (Table 1). All planned recordings were successfully acquired, with no missing data, and no recordings were excluded from the analyses. Baseline ABR latencies and amplitudes were consistent with stable intraoperative recordings (Table 2). After adjustment for averaging count, SR was not significantly associated with wave I, wave III, or wave V amplitude (all *p* > 0.7), suggesting that, in this dataset, rate-related effects were expressed primarily as latency changes rather than amplitude attenuation.

**Table 1:** Patient and recording characteristics.

Characteristic	Value
No. of cases	23
Age, y	56.78 ± 13.94，56.00 [49.00–65.00]
Sex	Women 10 (43.5%)，Men 13 (56.5%)
Diagnosis (TGN/HFS)	HFS 15 (65.2%)，TGN 8 (34.8%)
Side (R/L)	L 13 (56.5%)，R 10 (43.5%)
Ipsilateral pure tone average, dB HL	15.95 ± 8.38, 13.8 [7.5–23.18]
Contralateral pure tone average, dB HL	15.40 ± 8.07, 13.8 [9.1–20.98]
BIS	44.74 ± 7.62, 43.00 [40.00–49.00]
EtCO_2_, mmHg	40.35 ± 3.52, 40.00 [38.00–42.50]
Body temperature, °C	36.40 ± 0.40, 36.30 [36.20–36.65]
SpO2, %	98.83 ± 1.03, 99.00 [98.00–99.50]
Mean blood pressure, mmHg	65.04 ± 8.89, 66.60 [60.00–71.80]
Body weight, kg	66.76 ± 15.47, 67.60 [53.00–73.50]
Height, cm	165.51 ± 9.22, 164.00 [161.50–173.90]
TCI-Ce of propofol, μg/mL	2.49 ± 0.42, 2.40 [2.25–2.75]
Time to measurement, min	76.87 ± 11.78, 76.00 [69.50–83.00]
TIVA	yes 23 (100.0%)

Values are presented as mean ± SD and median [IQR] for continuous variables, and n (%) for categorical variables. EtCO_2_, end-tidal carbon dioxide; BIS, bispectral index; BT, body temperature; TCI—Ce, target effect-site concentration of propofol; TGN, trigeminal neuralgia; HFS, hemifacial spasm.

**Table 2:** Baseline auditory brainstem response parameters.

Parameter	Value (mean ± SD，median [IQR])	Unit
I wave latency	1.685 ± 0.126，1.680 [1.620–1.750]	ms
III wave latency	4.248 ± 0.235，4.240 [4.100–4.430]	ms
V wave latency	6.190 ± 0.375，6.220 [5.960–6.410]	ms
I–III interpeak latency	2.563 ± 0.216，2.600 [2.490–2.670]	ms
III–V interpeak latency	1.942 ± 0.252，1.900 [1.780–2.110]	ms
I wave amplitude	0.185 ± 0.214，0.270 [0.113–0.305]	μV
III wave amplitude	0.164 ± 0.223，0.192 [0.050–0.335]	μV
V wave amplitude	0.390 ± 0.274，0.420 [0.280–0.595]	μV

Baseline values were obtained at the lowest stimulus rate with the highest summation count. Values are presented as mean ± SD and median [IQR]. IPL, interpeak latency.

### 3.1 SR dependency of absolute wave latencies

Preoperative pure-tone audiometry showed a mean pure-tone average of 15.95 ± 8.38 dB in the ipsilateral ear and 15.40 ± 8.07 dB in the contralateral ear. Using directly measured latency values from the averaged ABR waveforms, linear mixed-effects models showed significant positive associations between SR and the absolute latencies of waves I, III, and V. Wave I latency increased by 0.00322 ms/Hz (95% CI, 0.00179 to 0.00465; *p* = 1.03 × 10^−5^). Wave III latency increased by 0.00843 ms/Hz (95% CI, 0.00672 to 0.01013; *p* < 0.001), and wave V latency increased by 0.00876 ms/Hz (95% CI, 0.00715 to 0.01037; *p* < 0.001). These findings indicate systematic rate-dependent prolongation of absolute latency across the ABR waveform, with larger effects observed for waves III and V than for wave I.

### 3.2 SR dependency of I–III and III–V IPL (Fig. 1, Fig. 2)

In the continuous mixed-effects model adjusted for averaging count (Table 3), SR showed a significant positive association with the I–III IPL. When SR was modeled as a continuous variable, each 1-Hz increase was associated with a 0.00521-ms prolongation of the I–III IPL (95% CI, 0.00307 to 0.00735; *p* = 1.75 × 10^−6^). For clinical interpretability, this coefficient corresponds to an estimated prolongation of 0.05211 ms per 10-Hz increase; this value represents a rescaling of the same continuous-model coefficient rather than a separate model.

**Fig. 1:**
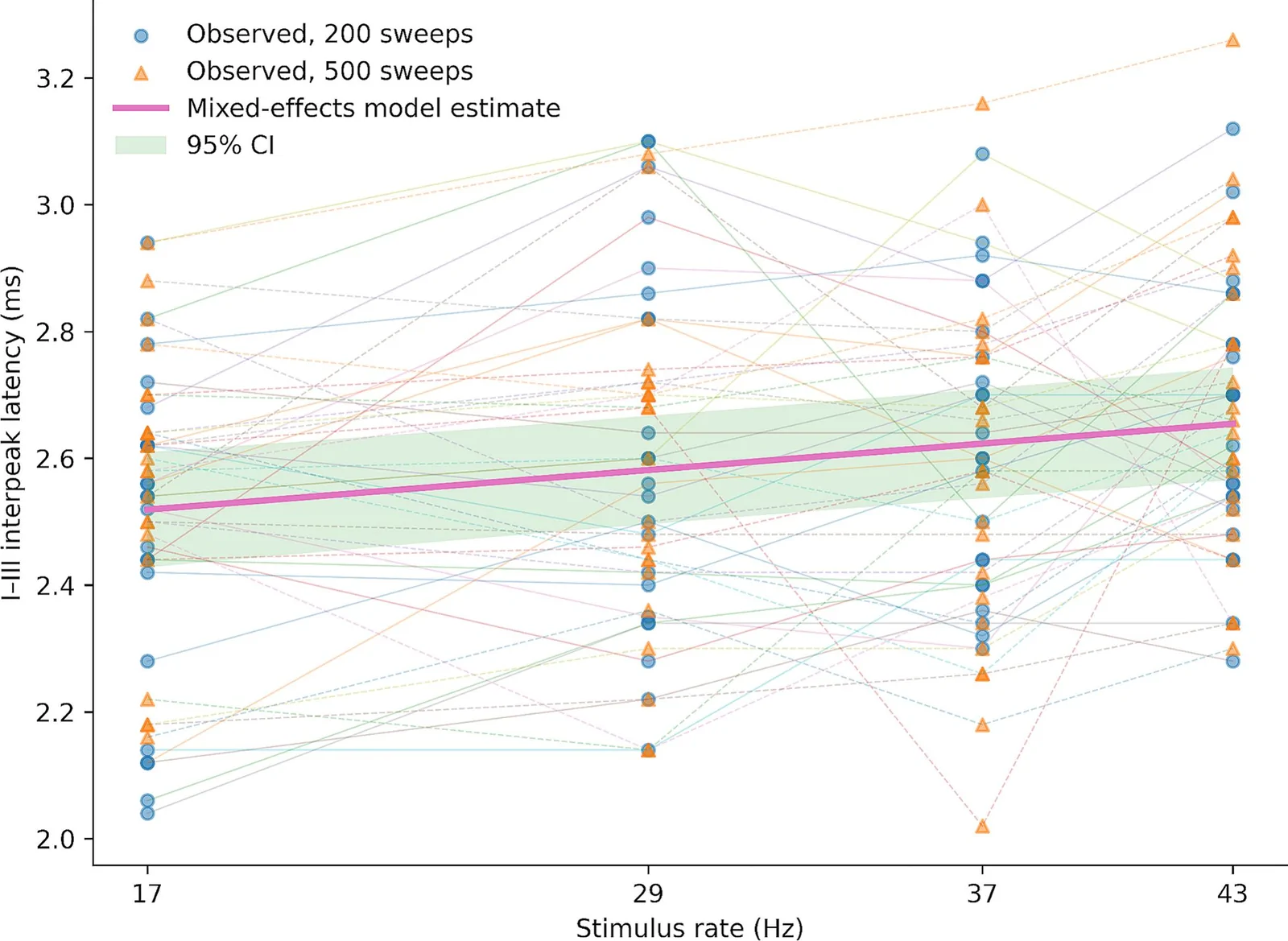
Stimulus rate–dependent prolongation of the I–III interpeak latency. Observed I–III interpeak latency (IPL) values are shown for each stimulus rate, with different symbols indicating recordings obtained with 200 and 500 sweeps. Thin lines represent within-patient trajectories of the observed measurements. The thick line represents the fixed-effect estimate derived from the linear mixed-effects model, and the shaded area represents its 95% confidence interval. The I–III IPL showed a significant positive association with stimulus rate when stimulus rate was modeled as a continuous variable (β = 0.00521 ms/Hz, 95% CI 0.00307–0.00735, *p* < 0.001). Averaging count was included as a covariate, and a random intercept for patient was included to account for repeated within-patient measurements. Model parameters were estimated using restricted maximum likelihood.

**Fig. 2:**
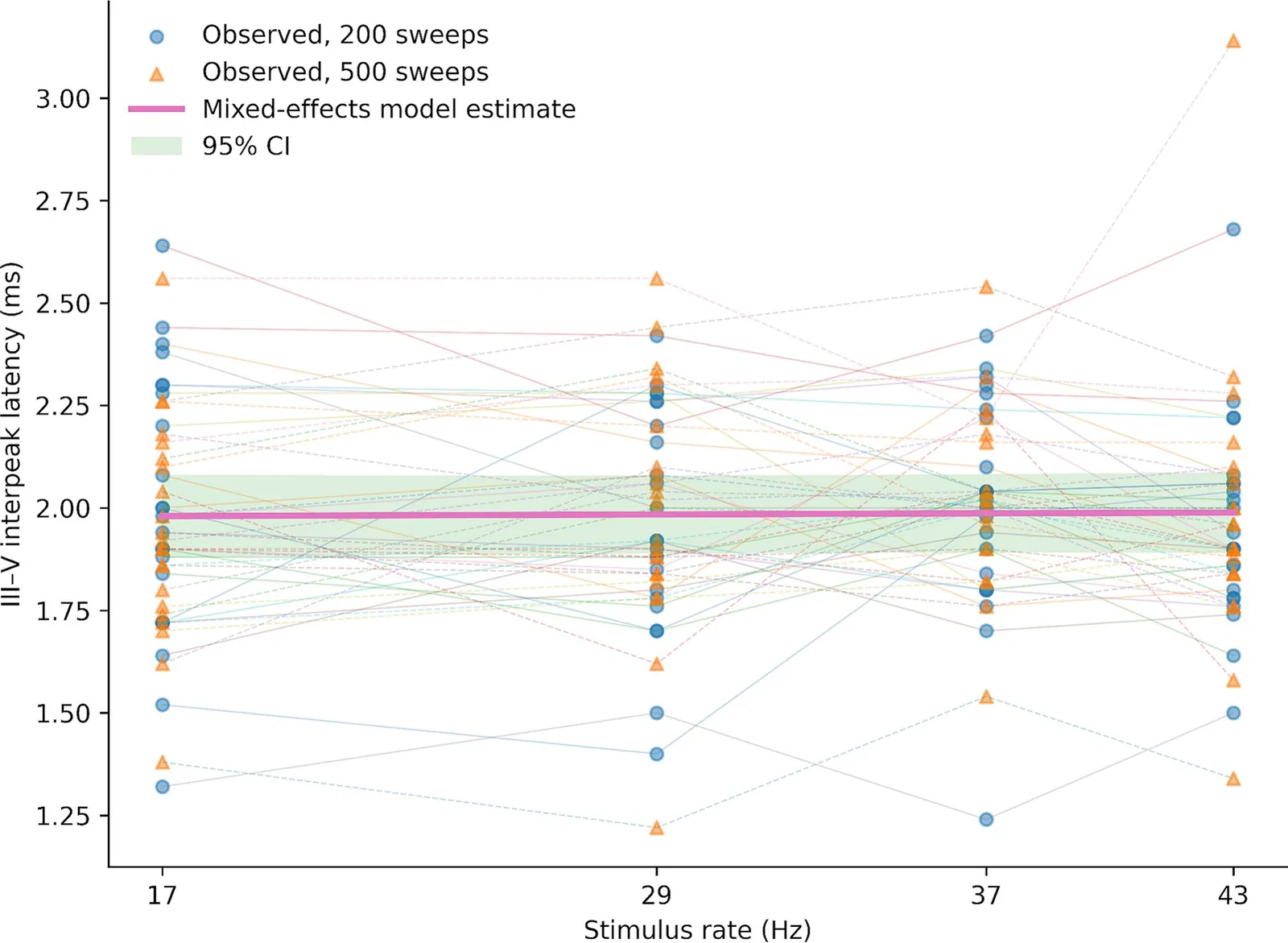
Absence of stimulus rate–dependent modulation in the III–V interpeak latency. Observed III–V interpeak latency (IPL) values are shown for each stimulus rate, with different symbols indicating recordings obtained with 200 and 500 sweeps. Thin lines represent within-patient trajectories of the observed measurements. The thick line represents the fixed-effect estimate derived from the linear mixed-effects model, and the shaded area represents its 95% confidence interval. No significant association between stimulus rate and the III–V IPL was observed when stimulus rate was modeled as a continuous variable (β = 0.00033 ms/Hz, 95% CI −0.00201–0.00267, *p* = 0.782). Averaging count was included as a covariate, and a random intercept for patient was included to account for repeated within-patient measurements. Model parameters were estimated using restricted maximum likelihood.

**Table 3:** Effect of stimulus rate on interpeak latencies (linear mixed-effects models).

Outcome	Effect	β (ms)	95% CI	95% CI	*P* value
			(lower)	(upper)	
I–III IPL	Continuous SR, per 1-Hz increase	0.005211	0.003074	0.007347	<0.001
I–III IPL	Continuous SR, per 10-Hz increase†	0.052108	0.030743	0.073473	<0.001
I–III IPL	29 Hz vs 17 Hz (reference)	0.068913	0.010661	0.127166	0.02
I–III IPL	37 Hz vs 17 Hz (reference)	0.071304	0.013052	0.129557	0.016
I–III IPL	43 Hz vs 17 Hz (reference)	0.152609	0.094356	0.210861	<0.001
I–III IPL	Categorical SR, overall (LR χ^2^ = 24.50, df = 3)	–	–	–	<0.001
III–V IPL	Continuous SR, per 1-Hz increase	0.000331	−0.002012	0.002674	0.782
III–V IPL	Continuous SR, per 10-Hz increase†	0.003307	−0.020125	0.026738	0.782
III–V IPL	29 Hz vs 17 Hz (reference)	0.012826	−0.051231	0.076883	0.695
III–V IPL	37 Hz vs 17 Hz (reference)	0.040435	−0.023622	0.104492	0.216
III–V IPL	43 Hz vs 17 Hz (reference)	−0.005652	−0.069709	0.058405	0.863
III–V IPL	Categorical SR, overall (LR χ^2^ = 2.35, df = 3)	–	–	–	0.503

Values are fixed-effect estimates from linear mixed-effects models with a random intercept for patient and averaging count included as a covariate. Continuous SR effects are presented per 1-Hz and per 10-Hz increase. †The per-10-Hz estimate is a rescaling of the coefficient from the same continuous model (β per 1 Hz × 10) and does not represent a separate model or a comparison with the 17-Hz condition. For categorical analyses, 17 Hz was used as the reference condition. LR, likelihood ratio; CI, confidence interval; IPL, interpeak latency; SR, stimulus rate.

When SR was modeled as a categorical factor, the overall effect remained significant (likelihood ratio [LR] χ^2^ = 24.497, df = 3, *p* = 1.97 × 10^−5^). Compared with the 17-Hz reference rate, the I–III IPL was significantly prolonged at 29 Hz (β = 0.06891 ms, *p* = 0.0204), 37 Hz (β = 0.07130 ms, *p* = 0.0164), and 43 Hz (β = 0.15261 ms, *p* = 2.82 × 10^−7^).

In contrast, the III–V IPL showed no significant association with SR (Table 3). In the continuous model, the slope was 0.000331 ms/Hz (95% CI, −0.00201 to 0.00267; *p* = 0.782), corresponding to 0.00331 ms per 10-Hz increase (95% CI, −0.0201 to 0.0267). Similarly, the categorical model showed no overall effect of SR (LR = 2.351; df = 3; *p* = 0.503).

As a sensitivity analysis, addition of a quadratic SR term did not significantly improve model fit for either the I–III IPL (*p* = 0.482) or the III–V IPL (*p* = 0.307), providing no evidence of a significant quadratic relationship over the tested SR range.

### 3.3 Efficiency optimization (time–stability trade-off)

An amplitude-weighted index combining acquisition time and latency stability was defined as follows:

$$E={\left\{\left(\mathrm{Sum}/\mathrm{SR}\right)\cdot SD_{V}^{2}\right\}}^{-1},$$

where *Sum/SR* represents the acquisition time, and *SD*_*V*_ denotes the SD of wave V latency within each SR–Sum condition. *SD*_*V*_ represents within-condition latency dispersion. Among the tested combinations (SR = 17, 29, 37, and 43 Hz; Sum = 200 or 500 sweeps), the highest efficiency was obtained at SR = 43 Hz with 200 sweeps, corresponding to an acquisition time of 4.65 s. This setting provided the most favorable balance between rapid acquisition and low wave V latency dispersion.

### 3.4 Extended optimization incorporating SR-dependent I–III prolongation

Because the continuous mixed-effects model estimated an average SR-dependent increase in the I–III IPL of 0.00521 ms/Hz over the tested range, the model-based expected shift relative to 17 Hz was defined as follows:

$$P\left(\mathrm{SR}\right)=k\left(\mathrm{SR}-17\right)$$

Based on this continuous-model estimate, the corresponding model-predicted shifts relative to 17 Hz were 0.0625 ms at 29 Hz, 0.1042 ms at 37 Hz, and 0.1355 ms at 43 Hz. To assess how tolerance for SR-induced I–III bias affected protocol selection, a sensitivity analysis was performed using allowable bias thresholds (δ) of 0.05, 0.10, 0.15, and 0.20 ms. For each threshold, the optimal condition was defined as the SR–Sum combination that maximized the amplitude-weighted index under the constraint P(SR) ≤ δ.

When δ = 0.05 ms, only 17 Hz satisfied the constraint, yielding an optimal setting of 17 Hz with 200 sweeps (acquisition time, 11.76 s). When δ = 0.10 ms, 29 Hz was the highest admissible SR because both 37 Hz and 43 Hz exceeded the 0.10-ms threshold, and the optimal condition shifted to 29 Hz with 200 sweeps (acquisition time, 6.90 s). When δ = 0.15 ms and δ = 0.20 ms, 43 Hz became permissible, and the optimal condition returned to 43 Hz with 200 sweeps, corresponding to the highest efficiency and shortest acquisition time (4.65 s). These findings show that the preferred SR depends strongly on the amount of I–III latency bias considered acceptable.

## 4 Discussion

This study demonstrated that, under standardized intraoperative TIVA conditions, SR preferentially affects early brainstem conduction, with little detectable effect on later conduction segments. The I–III IPL showed consistent and statistically robust SR-dependent prolongation, whereas the III–V IPL remained stable across the tested stimulation rates. These findings suggest that SR-related effects are not evenly distributed along the auditory brainstem pathway.

Physiologically, segments I–III reflect neural transmission from the distal auditory nerve to lower brainstem auditory relays, including the cochlear nucleus and superior olivary complex. Increasing SR may enhance neural adaptation, reduce phase synchrony, or increase temporal dispersion at these early relay stations. In contrast, the more rostral pathways contributing to the III–V IPL appeared comparatively less sensitive to SR under stable anesthetic conditions. Importantly, the I–III IPL showed a systematic SR-dependent prolongation that was well described by a linear model over the tested range and was consistent across patients, supporting a systematic rate-dependent effect rather than random measurement variability.

Linear mixed-effects modeling strengthened these conclusions by separating within-patient SR effects from between-patient variability. Standardized anesthetic management and consistent recording timing further reduced potential confounding, allowing clearer interpretation of rate-dependent conduction changes. Together, these findings suggest that SR selection during intraoperative ABR monitoring may selectively affect early conduction measures without a detectable effect on the later III–V segment.

### 4.1 Effects of Propofol and opioids on ABRs

The interpretation of the SR-dependent findings should be considered in the context of the known effects of anesthetic agents on ABRs. The influence of propofol on auditory-evoked responses has been examined in both adult and pediatric populations, with broadly similar conclusions.

In adult volunteers receiving nitrous oxide–supplemented anesthesia, propofol did not significantly alter brainstem ABR components. No dose-dependent changes were observed in the absolute latencies of waves I, III, or V or in the I–III, I—V, or III–V IPLs, whereas early cortical auditory evoked potentials showed concentration-dependent latency prolongation and amplitude attenuation (Savoia et al., 1988). Pediatric studies have reported modest but statistically significant dose-dependent prolongation of wave III and wave V latencies and related interpeak intervals during propofol anesthesia (Purdie and Cullen, 1993). However, the magnitudes of these changes were small and not clinically significant for diagnostic ABR interpretation.

Comparative studies have also suggested that propofol-based TIVA produces more stable brainstem responses than volatile anesthetics such as sevoflurane, which have been associated with greater latency prolongation (Kandil et al., 2022; Nakagawa et al., 2006). Taken together, the available literature suggests that, relative to inhalational agents, propofol has only limited effects on early brainstem conduction. Under standardized TIVA conditions, the observed SR-dependent modulation is therefore unlikely to be explained solely by anesthetic instability.

Remifentanil was administered concomitantly with propofol throughout ABR acquisition. Previous studies have suggested that remifentanil may modify middle-latency auditory evoked potentials (Schraag et al., 2006; Supp et al., 2018), whereas its effects on early brainstem auditory evoked potentials appear to be limited. In a study of healthy volunteers, remifentanil altered mid-latency auditory responses but produced no detectable changes in brainstem auditory evoked potentials within the first 10 ms after stimulation (Supp et al., 2018). Nevertheless, a potential contribution of the anesthetic regimen to absolute ABR latencies cannot be completely excluded. Because all SR conditions in the present study were recorded within each patient under the same standardized propofol–remifentanil TIVA conditions, any relatively stable anesthetic effect would be expected to influence the recordings similarly and is therefore unlikely to selectively account for the observed SR-dependent changes.

### 4.2 Mechanistic interpretation

The selective SR-dependent prolongation of the I–III IPL observed in this study is consistent with established rate-related changes in early ABR components. Experimental electrophysiological studies have shown that increasing SR prolongs early ABR latencies, likely reflecting neural adaptation and reduced synchrony within the auditory nerve and cochlear nucleus (Burkard and Hecox, 1983; Don and Eggermont, 1978).

Segments I–III encompass synaptic transmission in the cochlear nucleus, including the end bulb of the Held synapse. Experimental studies have shown that high-frequency stimulation can produce short-term synaptic depression and reduced neurotransmitter release at this level (Oleskevich and Walmsley, 2002; Wang and Manis, 2005). Such synaptic fatigue could contribute to cumulative micro-delays with increasing SR and may therefore provide a potential physiological explanation for the observed SR-dependent prolongation of the I–III IPL.

In contrast, segments III–V predominantly reflect transmission through more rostral auditory brainstem pathways, including the lateral lemniscus and inferior colliculus. These pathways may be less affected by rate-dependent synaptic depletion, and central resynchronization may reduce the impact of upstream temporal dispersion (Burkard and Hecox, 1983). This may explain the relative stability of the III–V IPL despite clear SR-dependent changes in the I–III segment.

However, this apparent stability should be interpreted with caution. Under TIVA conditions, later brainstem conduction may already be mildly prolonged at baseline, potentially reflecting anesthetic modulation of synaptic transmission and neural integration. As a result, the dynamic range for further SR-dependent prolongation in the III–V segment may be reduced, leading to an underestimation of rate effects. In contrast, the earlier segment (I–III), which is less affected at baseline, may retain greater sensitivity to SR. Accordingly, the absence of a significant SR effect in the III–V IPL does not necessarily indicate physiological resistance but may instead reflect a ceiling effect imposed by anesthesia-related baseline delay.

Overall, the present data are compatible with the possibility that the cochlear nucleus represents a rate-sensitive processing bottleneck within the auditory pathway under these physiological conditions.

### 4.3 Trade-off between acquisition efficiency and interpretability

The parallel prolongation model (Fig. 3) provides a conceptual framework for understanding the trade-off between acquisition efficiency and conduction stability. As SR increases, systematic delays accumulate in early auditory relays, resulting in predictable prolongation of the I–III IPL. Higher SR shortens acquisition time but introduces a measurable baseline shift in early conduction measures.

**Fig. 3:**
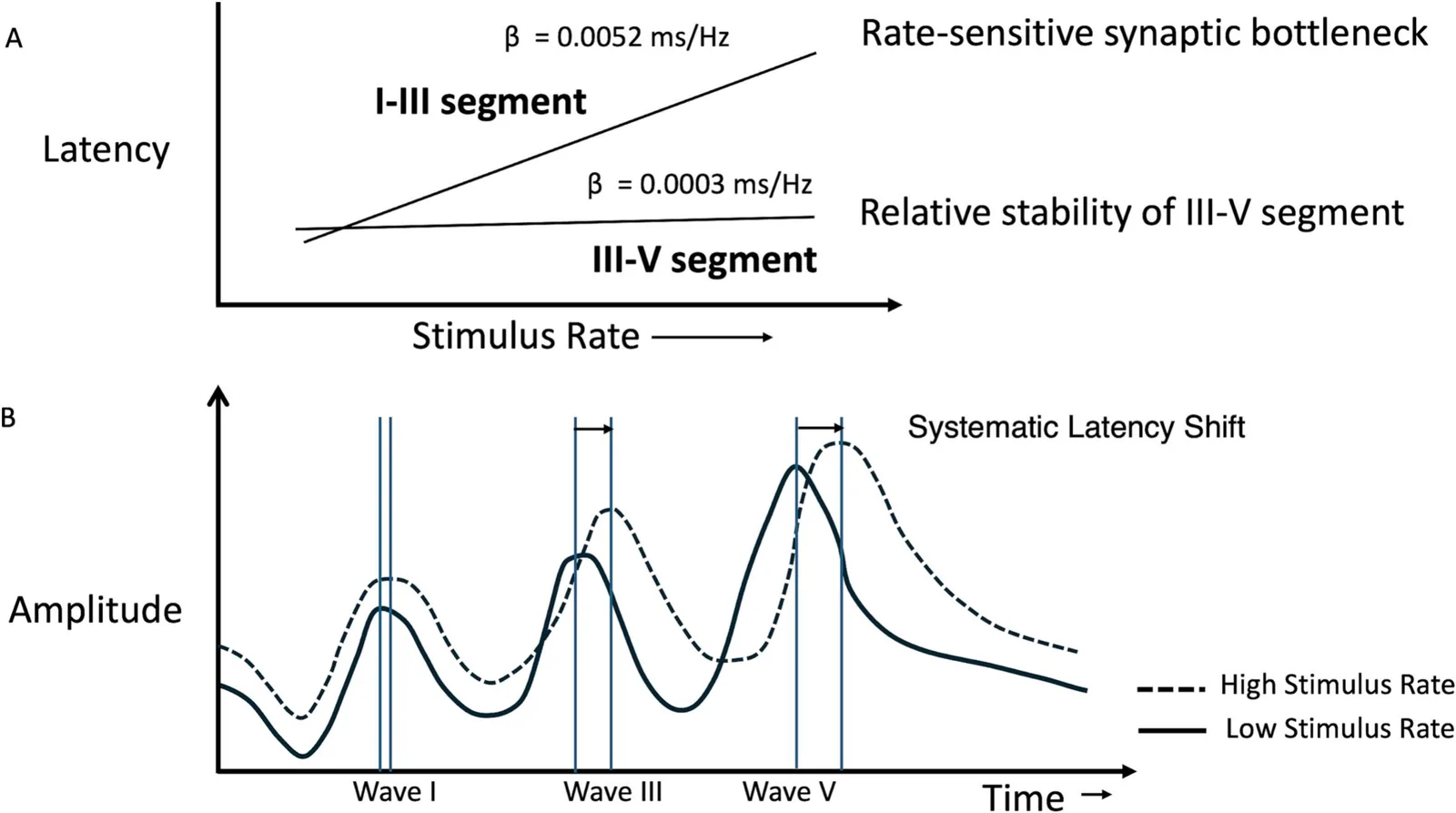
Optimization framework integrating acquisition efficiency and SR-dependent I–III latency bias. **(A)** Schematic representation of the segment-specific association between stimulus rate (SR) and auditory brainstem conduction observed in the present study. The I–III segment showed a significant positive association with SR when SR was modeled as a continuous variable (β = 0.0052 ms/Hz), whereas no significant association was observed for the III–V segment (β = 0.0003 ms/Hz). The “rate-sensitive synaptic bottleneck” shown in the schematic represents a proposed physiological interpretation of the observed I–III rate dependence rather than a directly demonstrated mechanism. The straight lines schematically illustrate the fixed-effect estimates from the continuous mixed-effects models over the tested SR range (17–43 Hz) and are not intended to imply an intrinsically linear physiological relationship beyond this range. **(B)** Schematic illustration of the effect of increasing SR on ABR peak latencies. The dashed waveform represents a higher-SR condition, and the solid waveform represents a lower-SR condition. Increasing SR is illustrated as producing a greater latency shift in the early I–III segment, with a comparatively limited additional shift in the III–V segment. The waveforms are conceptual representations intended to illustrate the observed segment-specific SR dependence and do not represent actual recorded ABR waveforms.

Sensitivity analysis indicated that SR selection should balance efficiency with tolerance for systematic I–III prolongation. At a lower SR (e.g., 17 Hz), the prolongation was minimal, but acquisition time increased. Intermediate rates (e.g., 29 Hz) may offer a practical compromise. At a higher SR (e.g., 43 Hz), acquisition efficiency was maximized at the cost of a predictable baseline shift. Importantly, this shift represents a systematic offset rather than random variability. When SR was maintained consistently throughout the monitoring period, relative intraoperative changes remained interpretable against a stable baseline. However, when absolute I–III thresholds are used as decision criteria, lower SR settings may enhance interpretability.

Therefore, high stimulation rates are not necessarily unsuitable for detecting subtle changes in ABR. Rather, rapid stimulation paradigms introduce predictable, segment-specific latency biases that may confound the interpretation of absolute IPL thresholds if they are not taken into account. In this framework, the main limitation of high SR lies not in reduced physiological sensitivity itself but in the presence of a systematic offset superimposed on true conduction changes. If this rate-dependent shift is recognized, rapid acquisition protocols may retain interpretability while providing better temporal responsiveness. Thus, the practical trade-off is less about speed versus sensitivity and more about speed versus uncorrected bias.

### 4.4 Implications for posterior Fossa surgery

In MVD and other posterior fossa procedures, prolongation of wave V latency remains a principal intraoperative warning sign of possible auditory pathway compromise (Hirono et al., 2014; Sindou et al., 2007; Ying et al., 2014). Loss or substantial delay of wave V has consistently been associated with postoperative hearing deterioration.

In addition, complete waveform loss, including disappearance of wave I, has been associated with more severe postoperative complications, such as profound hearing loss, dizziness, and tinnitus, suggesting a possible vascular-ischemic mechanism in advanced neural compromise (Joo et al., 2020). These observations suggest that waveform loss represents a late and often severe stage of auditory pathway injury rather than an early physiological disturbance.

Although I–III IPL prolongation has been associated with hearing outcomes in univariate analyses, it does not appear to be an independent predictor when overall ABR integrity is preserved (Thirumala et al., 2013). Nevertheless, early changes in wave III amplitude or latency may precede more global waveform changes. Disappearance of wave III has been reported as an early marker of surgical stress within the auditory pathway (Matthies and Samii, 1997).

In this context, SR-dependent baseline shifts in segments I–III warrant further investigation. Although such shifts are systematic and predictable, they may influence the interpretation of early segment changes during critical surgical stages. Therefore, recognition of this selective rate sensitivity may refine intraoperative decision-making and optimize monitoring protocols during MVD.

From a clinical perspective, although the SR-dependent prolongation of the I–III IPL was statistically significant and consistent across patients, its absolute magnitude was modest. Based on the continuous model, increasing the SR from 17 to 43 Hz corresponds to an estimated I–III IPL prolongation of approximately 0.14 ms. This degree of change is unlikely, by itself, to substantially alter the routine clinical interpretation of intraoperative ABR and should not be interpreted as indicating auditory injury or as requiring modification of current warning criteria. Rather, the present findings demonstrate a systematic SR-dependent shift that should be considered when selecting recording conditions and making precise longitudinal comparisons of ABR latency, particularly if the SR is changed during monitoring.

### 4.5 Limitations of this study

This study has several limitations. First, its retrospective design and relatively small sample size may limit the generalizability and stability of model estimates. Although repeated measurements across multiple recording conditions within each patient enabled assessment of within-patient SR-dependent changes, the limited sample size and single-center setting may restrict the external validity and generalizability of the findings. Validation in larger, prospective, multicenter cohorts is warranted. The restriction to MVD cases was intentional to minimize potential confounding from structural lesions involving or adjacent to the auditory pathway or brainstem. This relatively homogeneous cohort allowed us to evaluate SR-dependent changes under standardized conditions. However, the findings may not be directly generalizable to patients with intracranial tumors or other lesional pathologies, and validation in broader neurosurgical populations is warranted. Second, SRs were tested in a fixed ascending order with a short inter-recording interval; thus, potential order effects cannot be completely excluded. Third, although repeated measurements were accounted for using a mixed-effects model with a random intercept, random slopes were not included because of the limited sample size, which may have reduced the ability to capture inter-individual variability. Finally, the mechanistic interpretation of segment-specific latency changes is inferred from ABR patterns under intraoperative conditions and was not directly tested; therefore, these findings should be interpreted with caution. The present study was designed to characterize the effects of SR on ABR latency under stable conditions before skin incision and was not designed to evaluate postoperative auditory outcomes. Accordingly, the clinical significance of the observed SR-dependent latency changes in relation to postoperative hearing preservation remains to be determined. Further studies incorporating postoperative audiometric outcomes are needed before these findings can be translated into intraoperative warning criteria.

## 5 Conclusion

Under standardized intraoperative TIVA, the SR selectively modulated early auditory brainstem conduction while showing no detectable effect on the later conduction segment. The I–III IPL showed systematic rate-dependent prolongation over the tested stimulus-rate range, consistent with rate-sensitive processing in early auditory brainstem relays, including the cochlear nucleus. In contrast, the III–V segment remained comparatively stable across the tested stimulation rates. These findings suggest that SR introduces a predictable baseline shift in early conduction measures rather than random variability. Quantitative modeling of this rate dependence may improve the interpretation of intraoperative ABR monitoring, particularly in high-rate acquisition paradigms in which temporal responsiveness must be balanced against physiological interpretability.

## Ethics approval and informed consent

The study protocol was approved by the Ethics Committee of Chiba University Hospital. Written informed consent was obtained from all participants.

## Funding

This study did not receive any specific grants from funding agencies in the public, commercial, or nonprofit sectors.

## Declaration of generative AI and AI-assisted technologies in the manuscript preparation process

During the preparation of this work, the authors used ChatGPT to check grammar and improve readability prior to formal English editing. After using this tool, the authors reviewed and edited the content as needed and take full responsibility for the content of the published article.

## Declaration of competing interest

The authors declare that they have no known competing financial interests or personal relationships that could have influenced the work reported in this manuscript.
